# Atomic-Scale Insights into Phosphorene-Ionic Liquid Interface with Ab Initio Molecular Dynamics

**DOI:** 10.1021/acsphyschemau.5c00111

**Published:** 2025-12-29

**Authors:** Debora Ariana C. da Silva, Guilherme Colherinhas, Eudes Eterno Fileti

**Affiliations:** 1 Institute of Science and Technology, Federal University of ABC, Santo Andre, São Paulo 09210-170, Brazil; 2 Institute of Physics, Federal University of Goiás. Goiânia, Goiás 74690-900, Brazil; 3 Institute of Science and Technology, Federal University of São Paulo, São José dos Campos, São Paulo 12247-014, Brazil

**Keywords:** phosphorene, electrode ionic liquid interface, ab initio molecular dynamics, electric double layer, interfacial charge redistribution

## Abstract

The development of high-performance electrodes for supercapacitors and batteries remains hindered by an incomplete atomic-scale understanding of how material structure and polarization govern electric double-layer formation. In this work, we employ ab initio molecular dynamics (AIMD) simulations to probe the interface between a neutral phosphorene electrode and the ionic liquid EMIM-BF_4_, elucidating the mechanisms of charge redistribution and ionic ordering. Key findings include a detailed quantification of phosphorene’s structural flexibility, interplanar P–P distances averaging 0.224 and 0.231 nm with angular fluctuations up to 10°, and the characterization of a weak yet functionally significant electrode–electrolyte interaction energy of −138.2 kJ mol^–1^ nm^–2^ that drives pronounced interfacial ionic layering. Electron density and Hartree potential profiles reveal alternating regions of charge accumulation and depletion extending ∼2.5 nm from the surface, with local electric fields reaching 10^8^ V/m. Under zero bias, no appreciable charge transfer is observed, yet substantial local polarization effects underscore the critical role of the ionic liquid in modulating interfacial electrostatics.

## Graphical abstract

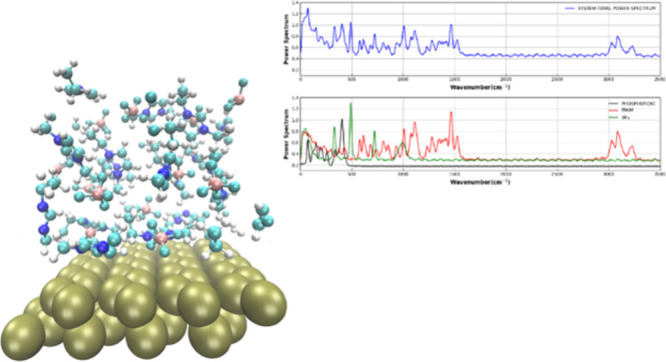

## 1 Introduction

Phosphorene, a two-dimensional form of black phosphorus, has emerged in recent years as a promising candidate for electrode materials in electrochemical energy storage systems due to its outstanding mechanical flexibility, moderate direct bandgap, and high electronic mobility.^1^ While black phosphorus has been known for decades, its exfoliated monolayer form—phosphorene—has gained increasing attention only recently, especially in the context of nanoelectronics and energy applications.^1,2^ This material exhibits a puckered and anisotropic structure that gives rise to a wide range of advantageous physicochemical properties, such as tunable electronic band structure, direction-dependent charge transport, and enhanced affinity toward adsorbed species.^3,4^ These characteristics have driven extensive research into its integration in devices such as transistors, photodetectors, and more recently, electrochemical energy storage technologies.^5–9^ Specific properties relevant for supercapacitor applications include its moderate bandgap, high carrier mobility, large surface area, and promising interactions with ionic species from electrolytes.^6–8^ However, one well-documented limitation of phosphorene is its sensitivity to environmental degradation.^10,11^ Studies report rapid oxidation in ambient conditions, which has a detrimental effect on both its structural integrity and its electronic performance in practical settings.^12^ This inherent chemical reactivity presents a central challenge for applications in which the material must remain stable in contact with reactive electrolytes over prolonged operation.

Recent theoretical studies using density functional theory (DFT) have examined the stability and adsorption behavior of ionic liquid species—such as the cation EMIM^+^ and anion BF_4_^–^—on phosphorene and similar 2D materials.^13^ These works consistently demonstrate that adsorption is governed by noncovalent interactions, with both ionic species interacting simultaneously with the phosphorene surface. The resulting charge transfer, although moderate, has a measurable impact on the electronic structure and may play a role in tuning the interfacial capacitance.^13^ Nevertheless, these findings are largely based on static calculations and do not account for thermal motion or quantum-level dynamic effects at the interface. Conversely, ab initio molecular dynamics (AIMD) has recently been used to model ionic liquid organization and polarization behavior under realistic conditions.^14,15^ These studies highlight the importance of considering polarization and charge redistribution in condensed phase systems, showing that ionic liquids such as EMIM-BF_4_ exhibit reduced effective ionic charges in solution and strong environmental dependencies.^15^ However, despite the relevance of AIMD to modeling dynamic processes at solid–liquid interfaces, no study to date has applied this method specifically to the phosphorene/EMIM-BF_4_ interface under conditions relevant to supercapacitor operation.

In this work, we employ AIMD simulations to explore the structural, electronic, and spectroscopic properties of monolayer phosphorene in direct contact with the ionic liquid EMIM-BF_4_, a system of significant relevance to supercapacitors and other electrochemical devices. As comparative references, we also analyze the behavior of both isolated materials in vacuum. This approach enables the quantification of interfacial charge redistribution, ionic interactions, and solvent-induced modifications to phosphorene’s properties, illuminating the role of quantum-level dynamics in the behavior of these emerging energy materials.

## 2 Methods

AIMD simulations were conducted to explore the electrical double layer properties of the ionic liquid EMIM-BF_4_ (1-Ethyl-3-methylimidazolium tetrafluoroborate) in interaction with phosphorene electrode. The studied systems consist of a phosphorene sheet interfacing with a segment of the ionic liquid electrolyte, sufficiently large to encompass the structured layers of the liquid. This approach (refer to Figure 1 and Table 1) includes all significant interactions at the surface of an uncharged phosphorene electrode. The simulation cell extends 3.5 nm along the surface normal, a value consistent with prior AIMD studies of ionic-liquid/2D-material interfaces. In this setup, the number-density profiles, Δρ­(z), and Hartree potential converge to bulk-like constant values beyond z ≈ 2.5 nm, confirming that a bulk electrolyte region is recovered within the chosen cell height. To establish baseline values, separate investigations were performed for the isolated components: phosphorene, the EMIM-BF_4_ ion pair, and the pure ionic liquid.

**Figure 1:**
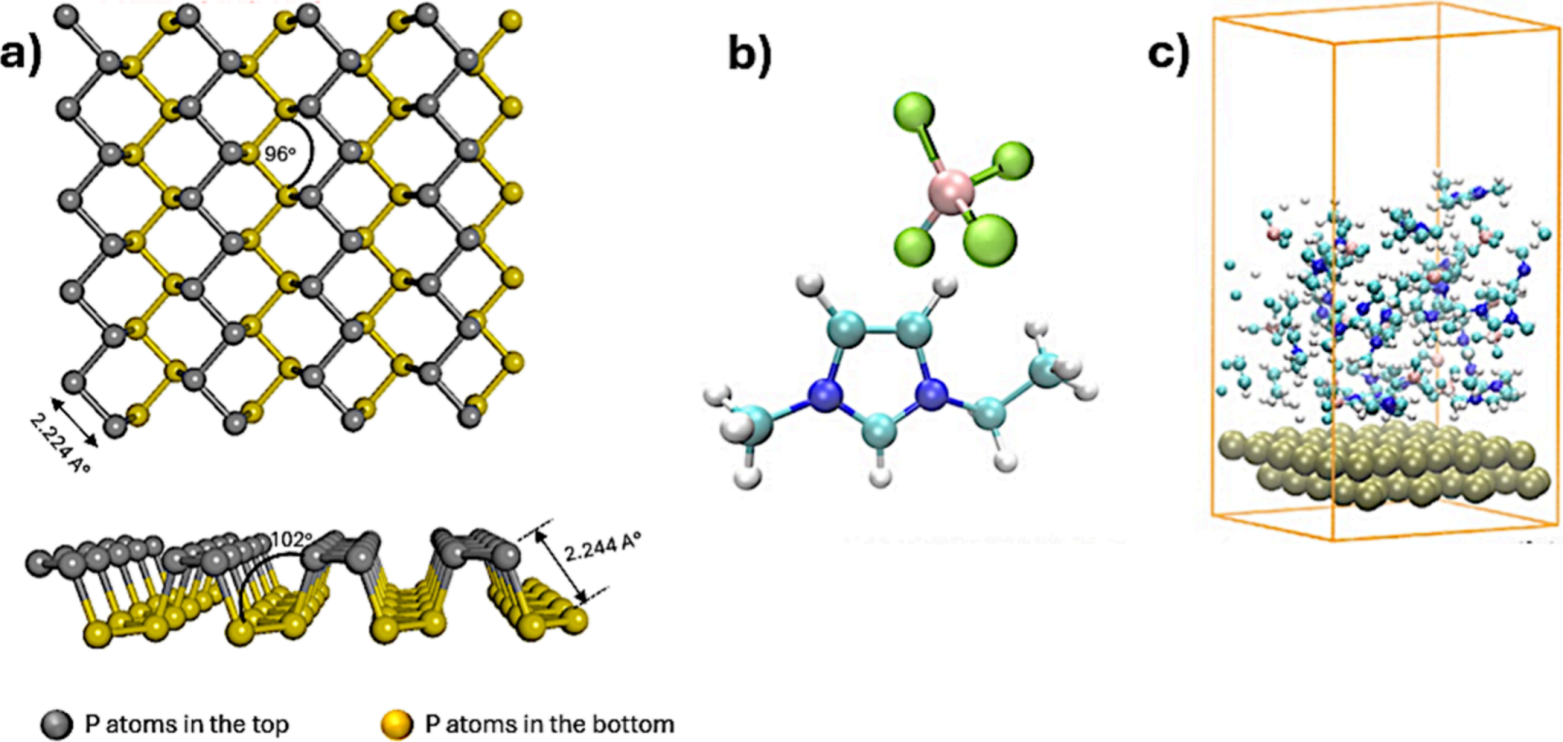
Molecular configurations of the systems studied. Top: (a) Phosphorene (b) EMIM-BF4 ion pair, and (c) a representative simulation cell.

**Table 1:** Specifications of the Simulated Systems, including the Number of Ion Pairs (#IP), Total Atom Count (#atoms) and Dimensions of the Computational Cell (X; Y; Z in pm)

system	#IP	#atoms	box size (pm)
phosphorene layer	0	80	1827; 1664; 3500
EMIM-BF_4_	16	384	1827; 1664; 3500
phosphorene + (EMIM + BF_4_)	16	80 + 384	1827; 1664; 3500

The simulation setups were generated using Packmol^16^ and equilibrated via classical molecular dynamics (CMD) simulations. The final equilibrated CMD configuration was adopted as the initial input for AIMD simulations. The AIMD time step was 0.5 fs, and total trajectory lengths were 40 ps for the phosphorene/ionic-liquid system and 20 ps for the isolated systems. All simulations were conducted in the NVT ensemble using a Nosé–Hoover chain thermostat, ensuring stable temperature (300 K) control throughout the trajectories. Electronic structures were computed using density functional theory (DFT) with the BLYP-D3 functional^17–19^ including Grimme’s dispersion correction^20^ and BLYP Goedecker-Teter-Hutter (GTH) pseudopotentials for core electron treatment.^21,22^ This functional was chosen for its capability to accurately describe noncovalent interactions,^23^ and its proven reliability in modeling ionic liquid structures and vibrational spectra.^24–26^ Additionally, it has been extensively benchmarked for ionic liquids and is known to reliably reproduce their noncovalent structure, dynamics, and vibrational spectra. In addition, BLYP-D3 provides accurate interaction energies for dispersion-dominated interfaces and has been successfully applied in previous AIMD studies of ionic-liquid/solid interfaces, including 2D materials. These considerations make it particularly suitable for capturing polarization, hydrogen bonding, and weak induction effects at the phosphorene/EMIM-BF_4_ interface. The calculations employed a double-ζ basis set (MOLOPT-DZVP-SR-GTH)^27^ within the generalized gradient approximation (GGA). The plane-wave and relative cutoffs were set to 350 and 40 Ry, respectively, and the SCF convergence criterion was 10^–6^. To treat the nonperiodicity of the system along the surface normal, the Poisson solver with slab (2D) correction was employed, which suppresses spurious slab–slab interactions under periodic boundary conditions. All AIMD simulations were executed using the QUICKSTEP module40 of the CP2K software package.^28^ Trajectory analyses were performed with TRAVIS^29^ and GROMACS tools.^30^

## 3 Results and Discussion

One of the main characteristics of phosphorene, which is highly desirable for application as an electrode in supercapacitors and batteries, is its large specific surface area. Considering the two atomic planes in the phosphorene monolayer, its specific surface area is estimated to be 1479 m^2^g^–1^, so for one plane, which will actually be in contact with the electrolyte, the area would be double that value, 2957 m^2^g^–1^. This value is 11% greater than that of graphene (2630 m^2^g^–1^),^31^ but phosphorene compensates for this with its intrinsic anisotropic structure and the presence of a direct bandgap, which enhances ion transport and energy storage capabilities. Moreover, the specific surface area of phosphorene can be further optimized through functionalization or exfoliation processes.^32^ These modifications not only improve its specific area but also enhance its stability and electrochemical performance in various energy storage systems.

Unlike graphene or MXenes, the internal structure of black phosphorus adopts a zigzag configuration, where sp^3^-hybridized phosphorus (P) atoms form covalent bonds, defining its characteristic framework. Consequently, phosphorus atoms can be found in two distinct atomic planes. Using AIMD simulations, we conducted a detailed assessment of the flexibility of phosphorene in vacuum by analyzing the standard deviation of key geometric parameters (including distance, angle, and dihedral distributions, as well as radial distribution and coordination number functions), as presented in Figure 2.

**Figure 2:**
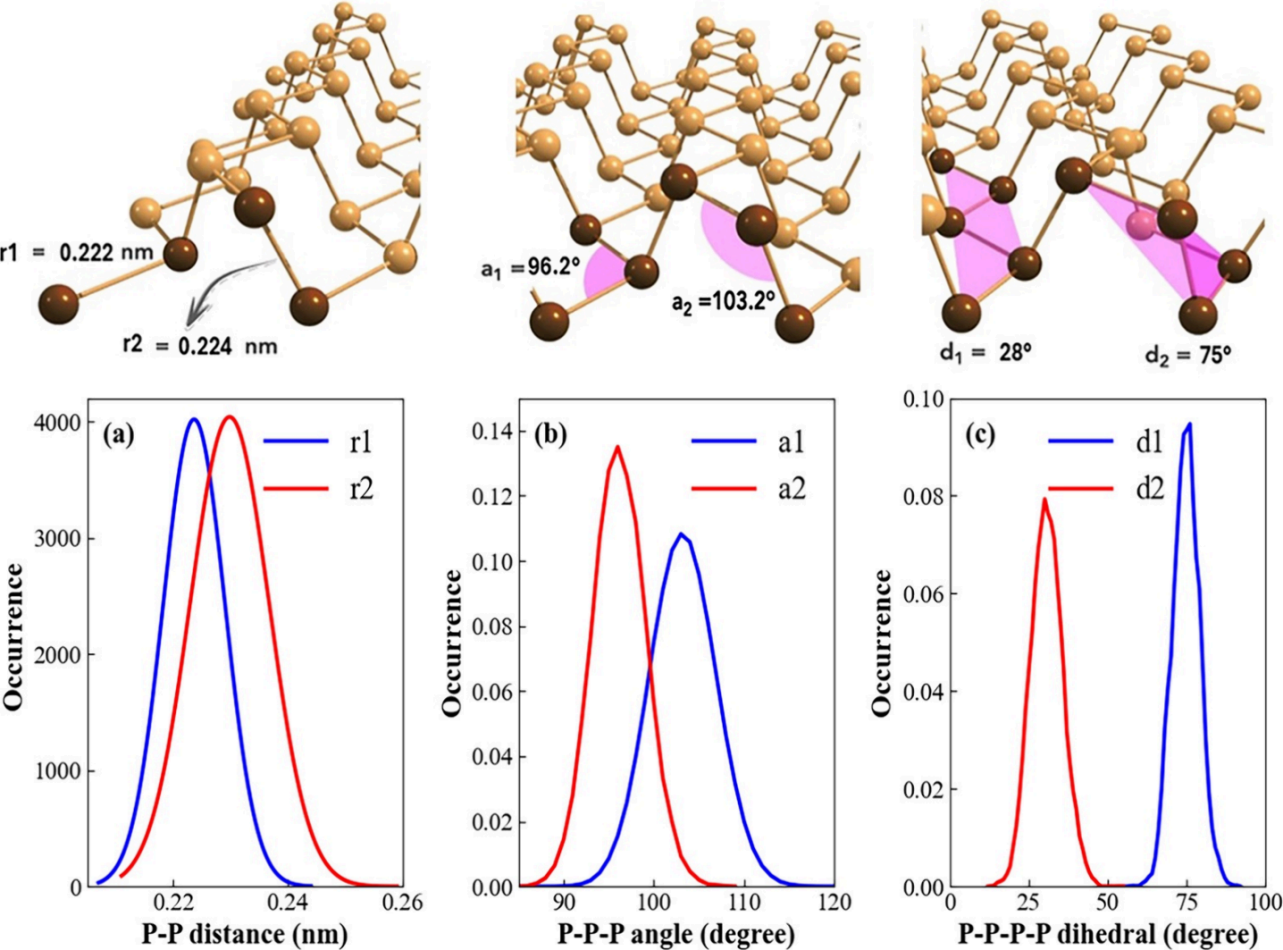
Structural properties of phosphorene electrode determined by AIMD. a) Interatomic distance between the ring atoms in and adjacent planes. b) angle between P–P bonds. c) dihedrals in and atoms in adjacent planes.

Phosphorene is characterized by two distinct interatomic distances: one connecting two atoms within the same atomic plane and another linking atom from different planes (see Figure 2a). Previous static DFT calculations, which do not account for thermal effects, report these distances as 0.222 and 0.224 nm, respectively.^33,34^ Our results, which incorporate dynamic fluctuations and thermal effects, yield corresponding distances of 0.224 and 0.231 nm. Notably, the standard deviation serves as a quantitative indicator of the structural flexibility of phosphorene, showing that both distances fluctuate within an amplitude of up to 0.02 nm.

Periodic static DFT calculations further reveal that the angle between two adjacent P–P bonds within the same atomic plane is 98.15°, while the angle between two adjacent P–P bonds in different atomic planes is 103.69°.^34^ In our AIMD simulations, these mean values (Figure 2b) were found to be 96.2° and 103.2°, with standard deviations indicating angular variations of up to ∼ 10° due to structural deformations.

To complete the analysis of phosphorene’s structural dynamics, we investigated two different dihedral angles: one describing torsional fluctuations within the same atomic plane and another related to torsion between atoms across adjacent puckered ridges. The values obtained, approximately 30.6° and 74.7°, are consistent with the intrinsic puckered geometry of monolayer phosphorene, as reported in structural models based on experimental studies.^35^

It is important to highlight that phosphorene exhibits high reactivity to oxygen and moisture, leading to oxidation and structural degradation.^10,36^ These characteristics compromise its integrity and electrochemical performance, particularly in systems exposed to open-air environments. For this reason, in this study, we selected a nonreactive electrolyte. As a result, throughout the entire simulation period, the phosphorene maintained its structural integrity, suggesting that this electrolyte may serve as a protective material, effectively mitigating the inherent degradation issues associated with phosphorene. This stabilization of phosphorene in contact with EMIM-BF_4_ arises from a combination of steric and chemical effects. The dense packing of cations and anions at the interface forms a physical barrier that limits direct access of reactive species to the surface, effectively providing steric shielding. In addition, both EMIM^+^ and BF_4_^–^ exhibit intrinsically low chemical reactivity toward phosphorene under zero-bias conditions, showing no tendency to form covalent bonds or trigger oxidative pathways during the AIMD trajectory. The joint action of interfacial ion packing and the benign chemical character of the ionic liquid explains why phosphorene remains structurally intact throughout the simulation.

The radial pair distribution function (RDF), depicted in Figure 3a, exhibits the characteristic behavior of ordered systems, with peaks appearing at well-defined positions. The first peak is observed at approximately 0.227 nm, followed by a second peak at 0.344 nm. Notably, the peak widths are not arbitrary; rather, they are intrinsically linked to the amplitude of atomic displacements within the electrode, which can only be discerned in a dynamic system. The integration of each RDF yields the coordination number as a function of interatomic distance (Figure 3b), revealing that within a radius of 0.8 nm, a given phosphorus atom randomly selected within the phosphorene structure is coordinated with approximately 50 neighboring atoms.

**Figure 3:**
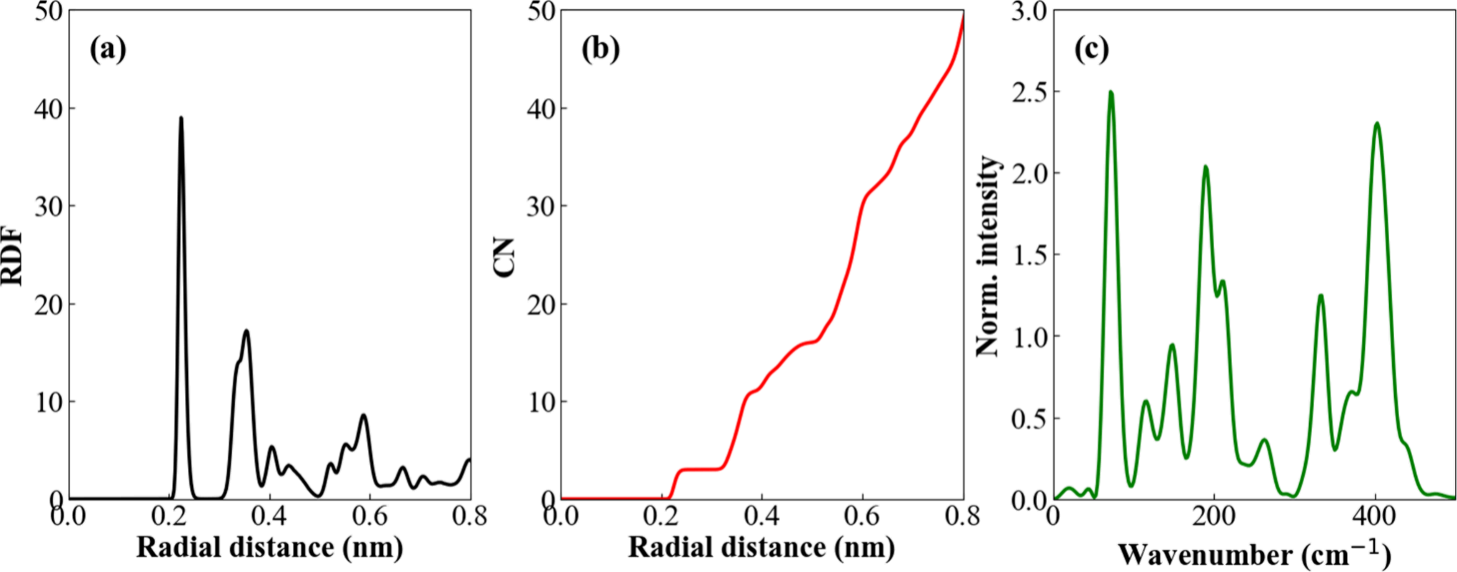
a) Planar radial distribution (xy) of pairs normalized by number density. b) Coordination number. c) Power spectrum of the phosphorene in the gas phase.

The vibrational behavior of the phosphorene can be characterized by the power spectrum, obtained by the Fourier transform of the velocity autocorrelation function of all atoms in the system.^37,38^ This spectrum includes all types of atomic movements (translational, rotational, and vibrational motions) and not just those that are IR or Raman active. Figure 3c shows the power spectra for phosphorus, where we can see that all the peaks are in the low-frequency region (<500 cm^–1^). This region typically characterizes the normal modes of vibration that project out of the electrode plane.^39,40^ According to experimental Raman spectrum (Figure 1b), the phosphorene nanosheet shows three prominent peaks at 354 cm^–1^, 433 cm^–1^ and 462 cm^–1^, which can be attributed to A1g (out of plane mode), B2g, and A2 g (in-plane modes), respectively. The power spectrum can be compared with IR and Raman spectra, but not directly, as it reflects all the vibrations of the system, whereas IR and Raman depend on specific vibrational activity criteria. This is what we observe in Figure 3c: although the power spectrum shows all the peaks in the low-frequency region, we notice that the peak positions are shifted compared to those in the Raman spectrum. Such differences may be due to vibrational selection rules, intermolecular effects, anharmonicity, and computational limitations of the simulation.

The uncharged phosphorene electrodes interact with the electrical double layer of EMIM-BF_4_ through van der Waals and induction/polarization interactions. We can obtain the intensity of these interactions, between the electrode and the electrolyte, by calculating the total energy of the system (*E*_*system*_) and subtracting from it the energies of the electrode in vacuum and of the pure electrolyte, ie.:

$$\Delta E=E_{system}-\left(E_{electrode}+E_{electrolyte}\right)$$ 01

The interaction energy is normalized by the projected surface area of the exposed phosphorene face (the xy-area of the simulation cell). Thus, we find that the electrode–electrolyte interaction energies for the system phosphorene/EMIM-BF4 is −138.2 kJ mol^–1^nm^–2^. For neutral electrodes, the strength of this interaction is relatively weak, being lower than the energy of an individual typical covalent bond strength. As a result, it does not significantly impact the electrode’s structure or the electrolyte’s ions. However, this interaction with the electrolyte plays a crucial role in organizing and reorienting the ions on the electrode’s surface.

The structure of the electrical double layer can be analyzed through the number density distribution profiles of the system’s components, as illustrated in Figure 4. Panel 4a corresponds to pure electrolyte slices, serving as a reference where we observe that the outermost ions in the layers interact directly with the vacuum. As a result, the slice as a whole does not exhibit any specific structural order. Conversely, in the presence of the electrode (Figure 4b), a pronounced restructuring of the cations is evident, along with a lesser but noticeable rearrangement of the anions. This restructuring is characterized by a sharp peak in the number density distribution near the contact surface, signifying the interfacial structural ordering of these species. The internal structure of phosphorene is also reflected in this graph, where a distinct double peak (black curve) represents its two atomic layers, separated by a distance of approximately 0.2 nm. This separation is consistent with the P–P interlayer spacing shown in Figure 2a. Additionally, an asymmetry in the atomic distribution dynamics within the phosphorene plane is observed, marked by a small peak on the left side at the base of the distribution. This peak corresponds to the outward displacement of phosphorus atoms from the structure, a phenomenon that is absent on the opposite side of the distribution. The number density profiles for specific atomic species (Figure 4c) reveal trends similar to those observed for the center-of-mass distributions, with pronounced peaks at the electrode surface. All number-density profiles are normalized by volume (nm^–3^), ensuring direct comparability across species and systems. The small asymmetric peak observed on the low-z side of the phosphorene distribution originates from slight outward displacements of a subset of surface phosphorus atoms. These fluctuations reflect thermally induced rippling of the puckered lattice and are mildly enhanced by weak, transient interactions with nearby ions of the ionic liquid. While subtle, this feature is fully consistent with the intrinsic structural flexibility of phosphorene and persists as a low-amplitude contribution throughout the trajectory. Notably, the fluorine atoms of the anion and the hydrogen ring atoms of the cation exhibit the strongest interactions with the electrode.

**Figure 4:**
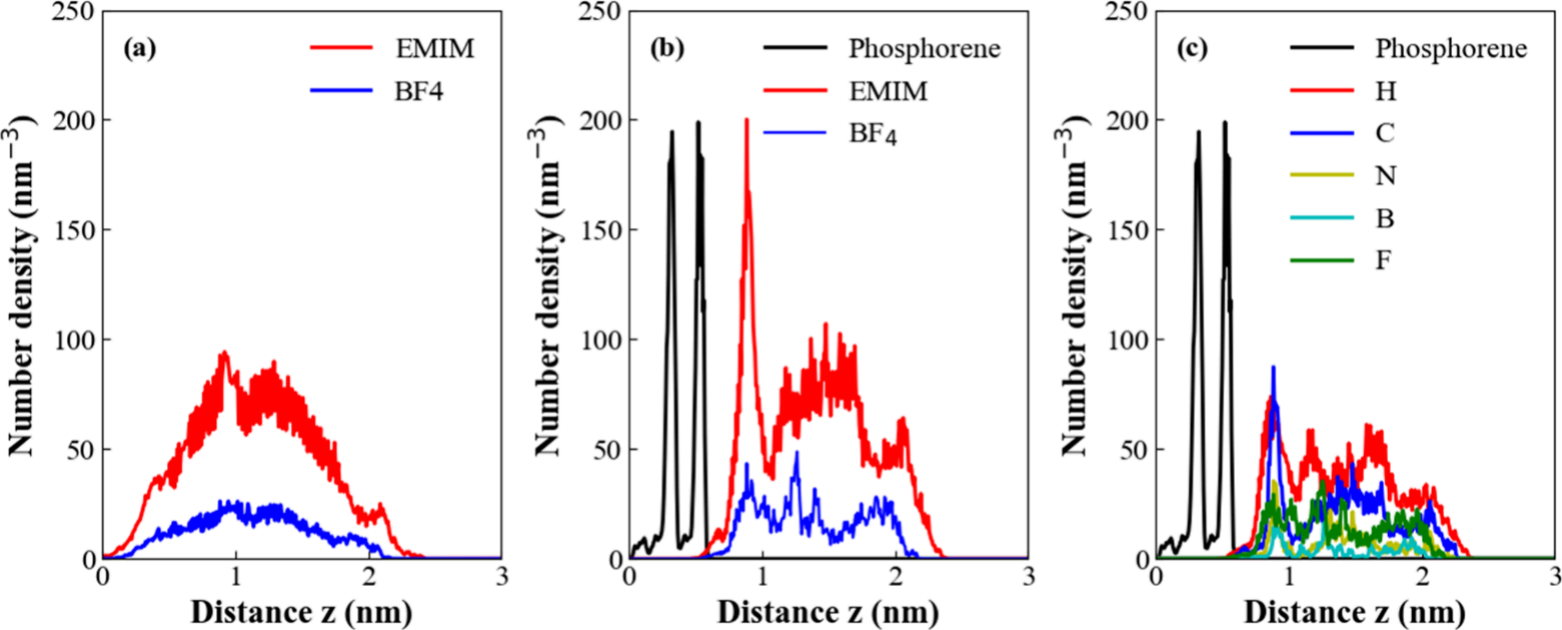
Number density profiles in nm^–3^. a) are the EMIM (blue) and BF_4_ (red) profiles to pure liquid slabs. b) are the EMIM and BF_4_ profiles for phosphorene system. c) are the selected atom-profiles for phosphorene system.

The intramolecular structure of the system can be elucidated through an analysis of its power spectrum. Figure 5 presents both the total and partial spectra for each investigated system. The global spectrum spans a broad frequency range, extending from 0 to 1600 cm^–1^, with an additional high-frequency region observed between 3000 and 3250 cm^–1^. The distinct vibrational signatures of each system can only be definitively assigned by comparing the global spectrum (Figure 5a) with the corresponding partial spectra (Figure 5b). This comparison reveals, for instance, that the high-frequency band is attributable to the stretching vibrations of the C–H bonds in the EMIM cation. Furthermore, all spectral features within the 1000–1600 cm^–1^ range correspond to the cation’s stretching modes. The BF_4_ anion exhibits well-defined and distinct peaks at approximately 350, 500, 750, and 1000 cm^–1^. In the low-frequency region, the vibrational modes of both the cation and anion overlap with those of phosphorene, whose intrinsic modes, as previously established, are confined to frequencies below 500 cm^–1^. A direct comparison between the spectrum of pristine phosphorene (Figure 3c) and the partial spectrum obtained in the presence of the electrolyte reveals no significant frequency shifts in these modes. This finding aligns with the weak interaction energy between the electrode and the electrolyte.

**Figure 5:**
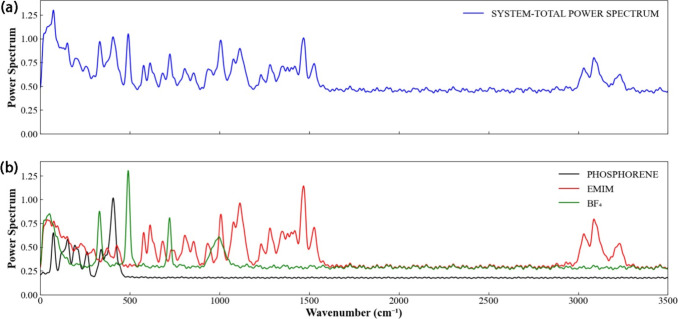
a) Total Power Spectrum for phosphorene/EMIM-BF_4_ system. b) Partial Power Spectrum for phosphorene, BF_4_ and EMIM. The spectra are normalized to the highest peak from the phosphorene spectrum.

To gain a deeper understanding of the changes in electron density distribution at the phosphorene/EMIM-BF_4_ interface, we mapped the electron density difference, as defined by the expression:

$$\Delta \rho =\rho_{system}-\left(\rho_{electrode}+\rho_{electrolyte}\right)$$ 02

As shown in Figure 6, we observe that the redistribution of electron density primarily occurs between the EMIM cations and the nearest atomic layer of phosphorene. In this particular configuration, we note the induction of both negative and positive charges in different regions of the electrode. This phenomenon appears to be the result of structural oscillations within the electrodes, and a more detailed analysis of the induced charge would require considering a configurational average over the entire trajectory. The observed redistribution is of small magnitude (the isodensity surfaces are within the range Δρ = ± 0.0002 e nm^–3^), and as expected, it does not suggest strong interactions that would indicate the formation of covalent bonds. This finding is consistent with the previous results of Ers and colleagues, who investigated the graphene/EMIM-BF_4_ interface, focusing on polarization effects and charge redistribution on the graphene surface.^40^ Similarly, they did not observe any evidence of charge transfer between the ionic liquid and the electrode.

**Figure 6:**
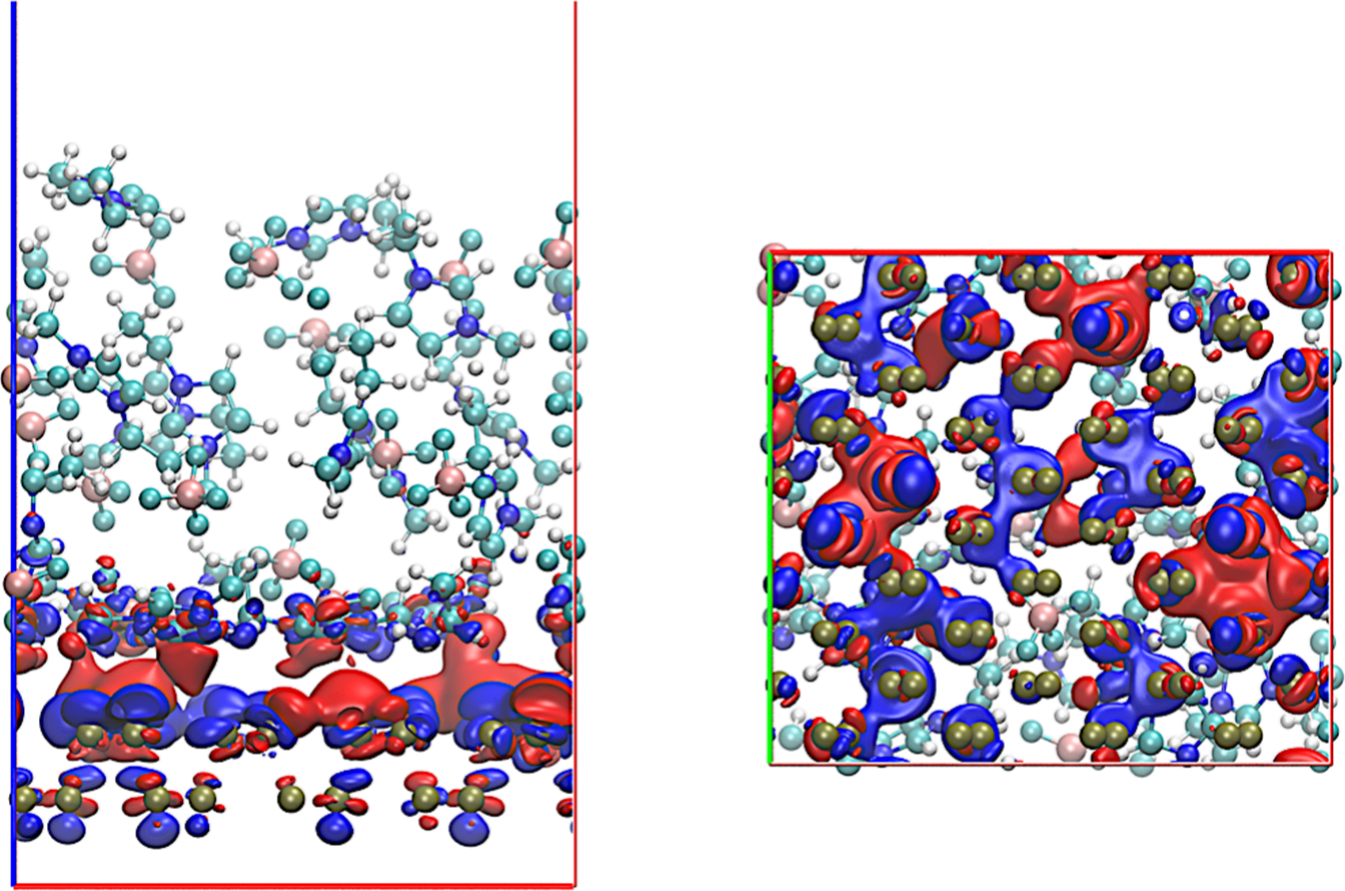
Electron density difference map at the phosphorene/EMIM-BF_4_ interface. The isosurface is represented with isovalues of Δρ = ± 0.0002e nm^–3^. Regions in red indicate electron density accumulation, while regions in blue indicate electron depletion. These charge redistributions reflect the interfacial interactions between the ionic liquid and the phosphorene surface, providing insight into local polarization effects and potential charge transfer at the interface.

To assess the robustness of the interfacial charge-redistribution picture, we verified whether the qualitative features observed in the electron-density difference map (Figure 6) persist across the AIMD trajectory. Although the figure displays a representative snapshot for visualization purposes, additional frames sampled throughout the simulation show the same overall pattern: localized polarization at the first ionic layer, absence of net charge transfer, and alternating regions of mild accumulation and depletion near the surface. While the precise magnitude and spatial extent of Δρ fluctuate due to thermal motion, the underlying qualitative behavior is fully reproducible across the ensemble, confirming that the observed interfacial polarization is not an artifact of a particular configuration but a persistent feature of the neutral phosphorene/EMIM-BF_4_ interface. It is important to note that these results were obtained for neutral electrodes in the absence of any external potential, meaning that charge transfer is minimal under zero bias. However, this scenario could change significantly if the systems were externally charged, allowing for a greater degree of local fluctuation in the field strength, potentially leading to the dispersion of local fields, driven by the dynamics of the electrolyte solution. To elucidate these effects, as well as other catalytic properties of phosphorene, further analyses involving electrode charging and the application of external potentials will be necessary. These investigations will be addressed in future work.

Figure 7 provides a characterization of the charge-density profile Δρ­(z) and the Hartree potential, V_H_(z) along the axis normal to a phosphorene surface immersed in EMIM-BF_4_. The Δρ­(z) profile (Figure 7a) maps the electronic redistribution within the electric double layer: a pronounced negative peak at ∼ 0.6 nm indicates electron depletion from phosphorene, followed by well-defined alternating accumulation and depletion layers corresponding to the ordered EMIM^+^ and BF_4_^–^ ionic layers. These oscillations delineate the effective thickness of the double layer and the decay length of the electrostatic perturbation, which vanishes beyond ∼ 2.5 nm as Δρ­(z) returns to zero, marking the onset of bulk-like electrolyte behavior. The Hartree potential (Figure 7b) quantifies the resulting potential drop and local electric field. The deep minima at ∼ 0.3 nm and ∼ 0.5 nm coincide with the most intense depletion regions in Δρ­(z), evidencing local fields on the order of 10^8^ V/m. Subsequent oscillations in V_H_(z) up to ∼ 2 nm mirror the ionic layering and illustrate the progressive screening of the electrode potential. In the bulk regime (*z* > 2.5 nm), V_H_(z) levels off, defining the reference potential. These electrostatic profiles, averaged over 100 trajectory snapshots, deliver essential insights for estimating differential capacitance, charge-transfer barriers, and the microscopic mechanism of electrode polarization in quantum-mechanical simulations of electrode–electrolyte interfaces.

**Figure 7:**
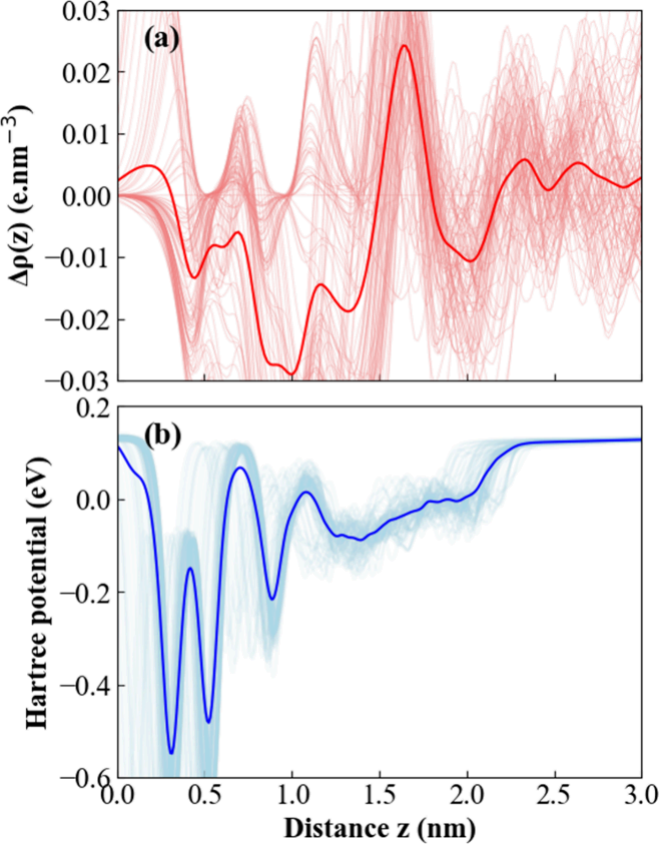
Electrostatic properties at the phosphorene/EMIM-BF_4_ interface. (a) Charge-density profile, Δρ­(z) (in e. nm^–3^), extracted from the electronic density as a function of distance z normal to the phosphorene electrode. (b) Hartree potential (in eV) along the same *z*-axis. Both curves represent ensemble averages over 100 distinct frames sampled from the AIMD trajectory, ensuring statistical convergence of the interfacial charge redistribution and electrostatic potential.

## 4 Conclusions

Through AIMD simulations grounded in DFT and rigorous trajectory analyses, we have demonstrated that phosphorene’s intrinsic structural anisotropy and flexibility facilitate a distinctive electric double-layer architecture when interfaced with EMIM-BF_4_. The electrode–electrolyte interaction, although energetically modest, orchestrates a tightly ordered ionic arrangement and induces significant local polarization without compromising the phosphorene lattice. Detailed mapping of electron density differences and electrostatic potential profiles uncovers oscillatory charge redistribution and field intensities on the order of 10^8^ V/m, decaying to bulk behavior beyond 2.5 nm. These insights not only clarify the fundamental interfacial phenomena governing differential capacitance and charge-transfer barriers but also establish a predictive framework for tuning phosphorene’s electrochemical performance. Future investigations will extend this methodology to charged electrodes and applied potentials, aiming to unlock enhanced charge-storage and catalytic functionalities.
